# ^1^H Nuclear Magnetic Resonance (NMR) Metabolomic Study of Chronic Organophosphate Exposure in Rats

**DOI:** 10.3390/metabo2030479

**Published:** 2012-07-24

**Authors:** Todd M. Alam, Muniasamy Neerathilingam, M. Kathleen Alam, David E. Volk, G. A. Shakeel Ansari, Swapna Sarkar, Bruce A. Luxon

**Affiliations:** 1 Department of Electronic and Nanostructured Materials, Sandia National Laboratories, Albuquerque, NM 87185, USA; Email: tmalam@sandia.gov (T.M.A.); 2 Department of Biochemistry and Molecular Biology, School of Medicine, University of Texas Medical Branch, Galveston, TX, 77555, USA; Email: baluxon@utmb.edu (B.A.L.); 3 Energetics Characterization Department, Sandia National Laboratories, Albuquerque, NM 87185, USA; Email: mkalam@sandia.gov (M.K.A.); 4 Institute for Molecular Medicine for the Prevention of Human Diseases, Centers for Proteomics & System Biology, University of Texas Health Science Center Houston, 1825 Pressler, Houston, TX 77555, USA; Email: David.Volk@uth.tmc.edu (D.E.V.); 5 Department of Pathology, University of Texas Medical Branch, Galveston, TX 77555, USA; Email: sansari@utmb.edu (S.A.)

**Keywords:** NMR, metabolomics, tributyl phosphate, triphenyl phosphate, chemometrics

## Abstract

^1^H NMR spectroscopy and chemometric analysis were used to characterize rat urine obtained after chronic exposure to either tributyl phosphate (TBP) or triphenyl phosphate (TPP). In this study, the daily dose exposure was 1.5 mg/kg body weight for TBP, or 2.0 mg/kg body weight for TPP, administered over a 15-week period. Orthogonal signal correction (OSC) -filtered partial least square discriminant analysis (OSC-PLSDA) was used to predict and classify exposure to these organophosphates. During the development of the model, the classification error was evaluated as a function of the number of latent variables. NMR spectral regions and corresponding metabolites important for determination of exposure type were identified using variable importance in projection (VIP) coefficients obtained from the OSC-PLSDA analysis. As expected, the model for classification of chronic (1.5–2.0 mg/kg body weight daily) TBP or TPP exposure was not as strong as the previously reported model developed for identifying acute (15–20 mg/kg body weight) exposure. The set of majorly impacted metabolites identified for chronic TBP or TPP exposure was slightly different than those metabolites previously identified for acute exposure. These metabolites were then mapped to different metabolite pathways and ranked, allowing the metabolic response to chronic organophosphate exposure to be addressed.

## 1. Introduction

Environmental exposure to organophosphates (OP) continues to be a concern due to the prevalent use of these chemicals in industrial applications. The identification of OP in almost every environmental matrix, including surface and ground water, air, soil, sewage and sludge, demonstrates the extent and persistence of these pollutants [1,2,3,4,5,6,7]. It has also recently been demonstrated that urinary metabolites of OP can be seen in the general human population at background levels [8]. Tributyl phosphate (TBP) and triphenyl phosphate (TPP), the focus of this paper, are used in aircraft hydraulic fluids and lubricant oils, flame retardant substitutes for halogenated compounds in plastics and resins, non-flammable plasticizers in acetate, polyester and polyurethane films, and antifoaming agents in concrete. TBP is also used during solvent extraction of nuclear waste and reprocessing of nuclear material based on the PUREX (Plutonium-Uranium Reduction Extraction) process. 

Numerous studies suggest that TBP and TPP are neurotoxic (delayed neurotoxicity) and may have possible teratogenic effects [9,10,11,12,13,14,15]. An overview of these findings is available from the International Programme of Chemical Safety (IPCS). On the other hand, the metabolism of TBP and TPP has seen fewer investigations. A ^14^C-labeling study showed that there are 11 different phosphate containing metabolites produced directly from TBP, with the mono- and di-butyl phosphates being the dominant metabolite species produced [18,19]. Additional studies identified sulfur containing metabolites, implying that glutathione-S-transferase is involved in the metabolism of TBP [18,19]. A single study involving the metabolism of TPP in liver homogenates determined that diphenyl phosphate is the primary product [20]. The metabolism of other organophosphates (primarily OP pesticides) commonly yields dialkyl phosphates, and as such these metabolites are used as biomarkers [21]. 

More recently, our group used nuclear magnetic resonance (NMR)-based metabolomics studies to explore correlating environmental exposure of TBP or TPP to changes in the urine metabolite profile of rats [22,23]. Metabolomic/metabonomics is a very powerful tool in determining the response of an organism to chemical intake or exposure. Metabolomics couples advanced spectroscopic detection techniques with multivariate or chemometric analysis to identify the metabolite signature associated with some environmental chemical exposure. Several excellent articles are available describing the use of NMR as applied to metabolites [24,25,26,27,28,29]. We have previously reported the metabolomics response of rats to an acute (one time) TBP or TPP exposure (TBP, 15 mg/kg body weight and TPP, 20 mg/kg body weight). In the case of TBP, there were three directly produced metabolites, dibutyl phosphate (DBP), *N*-acetyl-(S-3-hydroxybutyl)-L-cysteine and *N*-acetyl-(S-3-oxobutyl)-L-cysteine identified in the urine of treated rats [23]. In addition, it was shown that changes in the endogenous urinary metabolites could also be correlated with TBP exposure. A multivariate/chemometric analysis of the NMR spectra of urine from rats exposed to either TBP or TPP has also been reported. Using orthogonal signal correction (OSC)-filtered partial least squares discriminate analysis (OSC-PLSDA) a series of important metabolites were identified and ranked based on their ability to provide classification during the analysis [22]. The endogenous metabolites contributing to the exposure classification were taurine, betaine, 2-oxoglutarate, creatine and citrate; suggesting an impact on the citrate (TCA) cycle.

In this paper, we present an extension to these ^1^H NMR metabolomic studies of acute TBP and TPP exposure by evaluating the metabolomic response to a chronic, lower dose TBP and TPP exposure in rats over a 15-week period. The OSC-PLSDA method previously employed for the acute studies was also used here, thus providing a direct comparison of metabolites responsible for identification of acute and chronic organophosphate exposure.

## 2. Results and Discussion

### 2.1. ^1^H NMR of Urine Following Chronic TPP and TBP Exposure

**Figure 1:**
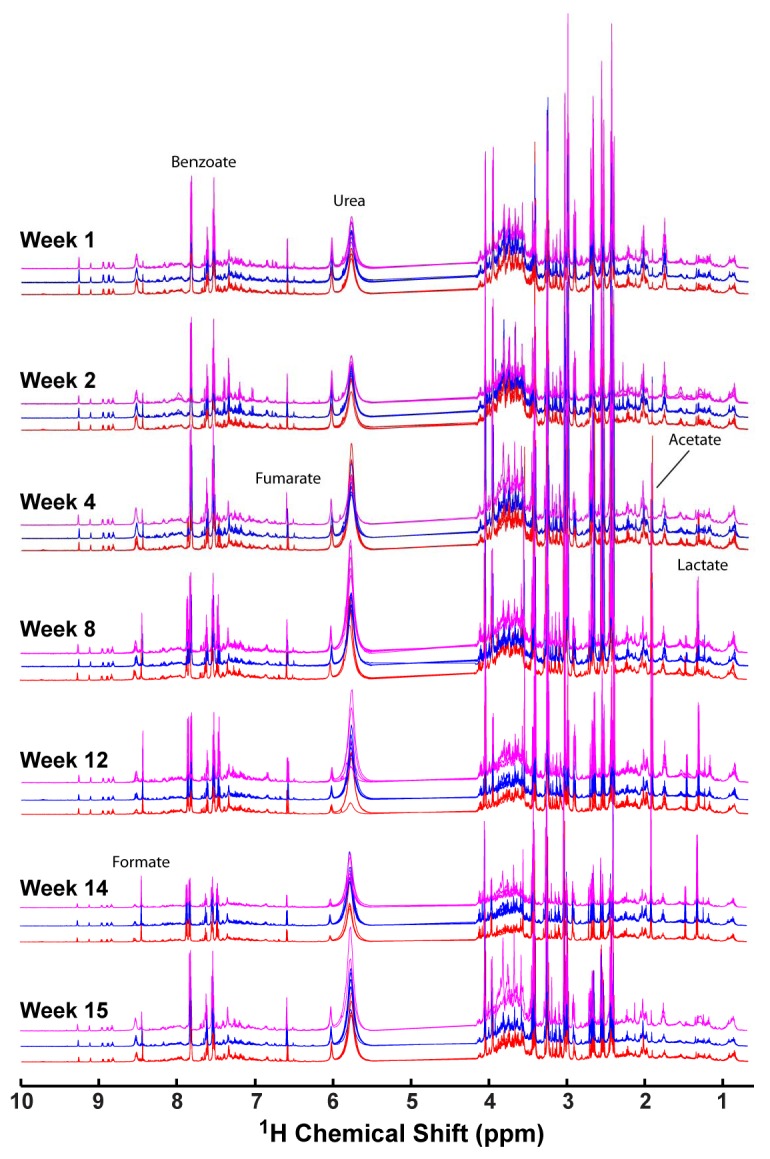
600 MHz ^1^H nuclear magnetic resonance (NMR) spectra of rat urine collected from control (blue), tributyl phosphate (TBP) (magneta) or triphenyl phosphate (TPP) (red) exposed animals over a 15 week period with chronic administered doses. The NMR spectra were referenced using the internal chemical shift indicator DSS (500 μM), with the overall signal intensity normalized using the quotient method. The water spectral region (4.1 to 5.5 ppm) was removed prior to analysis.

The normalized ^1^H NMR spectra of urine samples collected from TBP exposed (5 rats), TPP exposed (5 rats) and control animals (7 rats) for seven different time points during the total 15-week study are shown in Figure 1. As expected for urine samples, resonances for numerous metabolites are observed, with changes in the overall metabolite profile occurring as a function of both exposure time and exposure class. Even though previous studies of acute TBP and TPP exposure have identified specific spectral regions and metabolites that are impacted by exposure, we were unable to identify simple unique NMR spectral signatures that correlated with a given exposure class over all animals in that set. Note that the resonances previously assigned to the dibutylphosphate (DBP), an intermediate metabolic degradation species of TBP, would be observable at δ = +0.9 ppm. This spectral region does not reveal any significant intensity variation during the chronic exposure studies, suggesting that at this level of insult, the native detoxification mechanism(s) reduce the concentration of DBP below the NMR detection limits. There are also some metabolite spectral signatures that increase and then decrease during the exposure process, such as the singlet resonance at δ = +1.31 ppm, and the sharp singlet at δ = +1.91 ppm (Figure 1). Similarly, there are no large changes in the aromatic region that could be associated with the production of the diphenyl phosphate byproduct of TPP, again suggesting that the concentration of this metabolic degradation species is below NMR detection limits. To identify spectral regions that correlate with OP exposure a chemometric analysis of the entire exposure data set was undertaken, as described below. There are numerous multivariate methods that could be applied, including principal component analysis (PCA), soft independent modeling of class analogy (SIMCA), linear discriminant analysis (LDA), partial least squares (PLS), PLS-discriminant analysis (PLS-DA), along with non-linear methods such as hierarchical cluster analysis (HCA), self-organizing maps (SOMs), non-linear mapping (NLM) and genetic programming (GP) [29,30,31]. For this paper we have elected to utilize the same OSC-PLSDA method previously implemented in the analysis of acute TBP and TPP exposure [22], allowing direct comparison between the two studies.

### 2.2. OSC-PLSDA Model Development for Chronic TPP and TBP Exposure

Orthogonal signal correction (OSC)-filtered partial least squares discriminate analysis (OSC- PLSDA) was employed as previously described [32]. In this study, three classes were utilized: TBP-exposed (class 1), TPP-exposed (class 2) and control (class 3). The OSC-PLSDA method attempts to identify the spectral regions that are responsible for separation of the different classes and predicts the identity of each sample with a 1 designating a sample is included within a class, and a 0 if it is not being included in that class. During the development of the OSC-PLSDA model the number of latent variables (LV) required for classification needs to be identified. This was estimated by monitoring the change in classification error (% of sample misclassified) for each sample in the data set as a function of the number of latent variables. The classification error commonly drops quickly as the first few LV are incorporated into the model, followed by a slower reduction for higher number of latent variables. This allows the user to choose the number of latent variables to employ based on the magnitude classification error acceptable. For the present study we have chosen a classification error of <5% prior to cross validation. To prevent over fitting of the data set the minimum number of latent variables based on this error criteria was employed.

**Figure 2:**
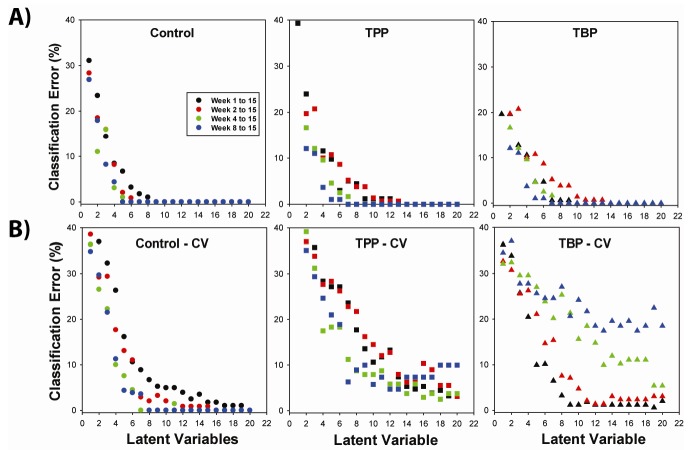
Classification error for identification of control, TPP- or TBP exposed rats as a function of the number of latent variables in the OSC-PLSDA model using the entire 15 week exposure data set. The error results are shown for **A**) the original model developed, and **B**) following cross validation (CV).

The classification error as a function of the number of LV for prediction of each exposure type during the 15-week chronic exposure is shown in Figure 2. The OSC-PLSDA model was originally evaluated using the NMR data from the entire 15-week sampling period (black symbols). Between six and eight LV were required to reduce the classification error below 5%, and in many instances produced perfect classification when using eight or more LV. The TBP classification error was slightly higher than the error for TPP or control classifications. The number of LV for this chronic model is similar to the six LV employed for the acute TBP and TPP exposure modeling [22], but for the acute model only six LV were required to obtain a classification error of zero (all samples correctly classified).

The classification errors obtained during cross validation (CV), using a venetian blind method (see experimental for details), are shown in Figure 2B and represents an average over all CV trails. There is clearly an increase in the error for all three classes, with a higher number of LV required to obtain the desired target 5% error level. For this chronic exposure NMR data set, between 10 and 14 LV were required to obtain classification errors below 5%. An exception is the identification of the TPP-exposed animals which hovers near 10% classification error (under CV) until over eighteen LV were employed. Table 1 provides a select summary of the classification errors with increasing number of LV. The high number of variables required for classification following chronic exposure is somewhat disappointing, and is in contrast to the six LV required to provide excellent classification in the acute exposure [22]. This result suggests that while the metabolic response to TBP and TPP exposure as monitored by ^1^H NMR analysis of the rat urine is present, it is not particular strong, nor is it an easily recognized response.

**Table 1:** Error of prediction for original model and after cross validation (CV) for the identification of control, TPP-exposed and TBP-exposed samples as a function of latent variables (LV) in the model and specific weeks evaluated.

System	% Error in Prediction (Model)				% Error in Prediction (Cross Validation)		
	Control	TPP	TBP		Control	TPP	TBP
**6 LV**	3.2	3.0	4.7		10.6	27.2	10.2
**8 LV**	1.7	4.4	0.6		6.7	17.7	3.2
**10 LV**	< 0.5	1.2	< 0.1		4.9	10.6	1.2
**12 LV** ***(Week 1 – 15)***	< 0.1	1.2	< 0.1		3.9	13.3	1.2
**12 LV** ***(Week 2 – 15)***	< 0.1	0.7	< 0.1		0.8	12.8	1.4
**12 LV** ***(Week 4 – 15)***	< 0.1	< 0.1	< 0.1		< 0.1	5.8	14.8
**12 LV** ***(Week 8-15)***	< 0.1	< 0.1	< 0.1		< 0.1	4.7	18.5

The question has been raised whether the exposure to TBP or TPP produces a delayed metabolic response, and if classification might be improved by only considering the NMR data response from later weeks? To explore this concern, the classification error was determined as a function of the number of latent variables (Figure 2) for subsets of the data involving weeks 2 through 15 (red), weeks 4 through 15 (green) and weeks 8 through 15 (blue). For classification of the control and TPP animals there is a slight improvement in the prediction error when only data from week 4 through 15 is included in the analysis, perhaps suggesting a delayed (but weak) metabolic response to TPP exposure. For example, the error in TPP prediction using 12 LV drops from 13.3 % to 5.8%, while the control classification error drops from 3.95 to <0.1%, by only evaluating the later exposure weeks. This delayed metabolic response was also observed in the variation of VIP scores as a function of time for the important metabolites (Figure S1, supplemental material), and discussed further in section 2.3. 

In contrast, the TBP classification error increases when analyzing data sets that include the later weeks of chronic exposure. For twelve LV, the TBP classification error jumps from 1.2% to almost 18.5% by going from the full (1 through 15 week) data set to a reduced (8 to 15 week) data set (see Table 1). This increasing prediction error with truncation of the first few weeks of exposure argues that for chronic TBP exposure, the metabolic changes that allow for classification are the strongest in the early weeks following TBP exposure. While it is possible to tailor the OSC-PLSDA model for optimal TPP or TBP classification by altering the sampling subset following exposure, we have elected to utilize the entire data set for the remaining analysis discussed below.

**Figure 3:**
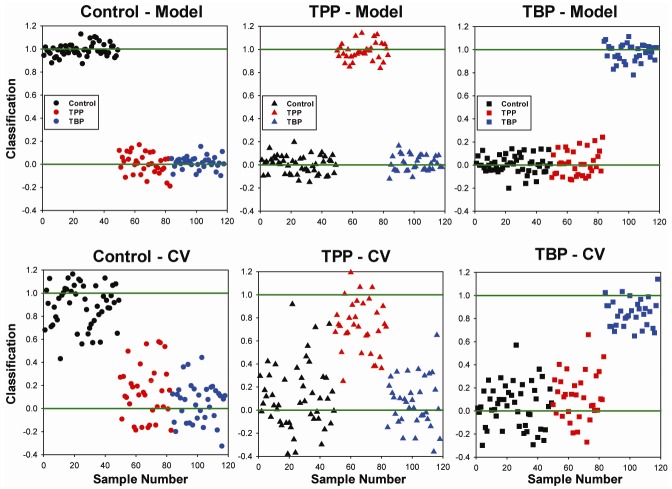
Prediction for sample identification into either the control, TPP-exposed or TBP-exposed class using the entire 15 week exposure data set, and 118 samples. The results for **A**) the original model involving 12 latent variables, and **B**) following venetian blind cross validation (CV).

Figure 3 shows the OSC-PLSDA classification results using twelve latent variables on the complete 15-week data set. For the original model, the separation of the three classes is excellent with all 118 urine spectra being correctly classified (classification errors < 1%, Table 1). A perfect classification score of 1.0 (positive) or 0.0 (negative) is shown as a green line in Figure 2 to provide a visual reference. Under cross validation there is an increased scatter in the error observed (Figure 3B) with some samples being misclassified (classification score < 0.6). The TPP classification errors reveal the greatest degree of scatter, which is consistent with the prediction errors shown in Table 1.

The data in Figure 3 is also grouped in time series for each exposure type. For example, samples 1 – 49 represent control rats (no TBP or TPP exposure), with week one on the left ending with week 15 on the right. Samples 50-83 are from the TPP exposed rats, with week one on the left and week 15 on the right. Similarly, urine samples 84–118 are from the TBP exposed animals, with week one on the left and week 15 on the right. Inspecting the time variation in the predicted classification score within each group (left to right), there were no large increases/decreases in the performance of the model with time observed. This again supports the argument that there were not any delayed metabolic responses to chronic exposure that become dominant in controlling the classification, and that the entire data set over the exposure period should be employed in the analysis.

### 2.3. Important Metabolite Identification Using VIP Scores

**Figure 4:**
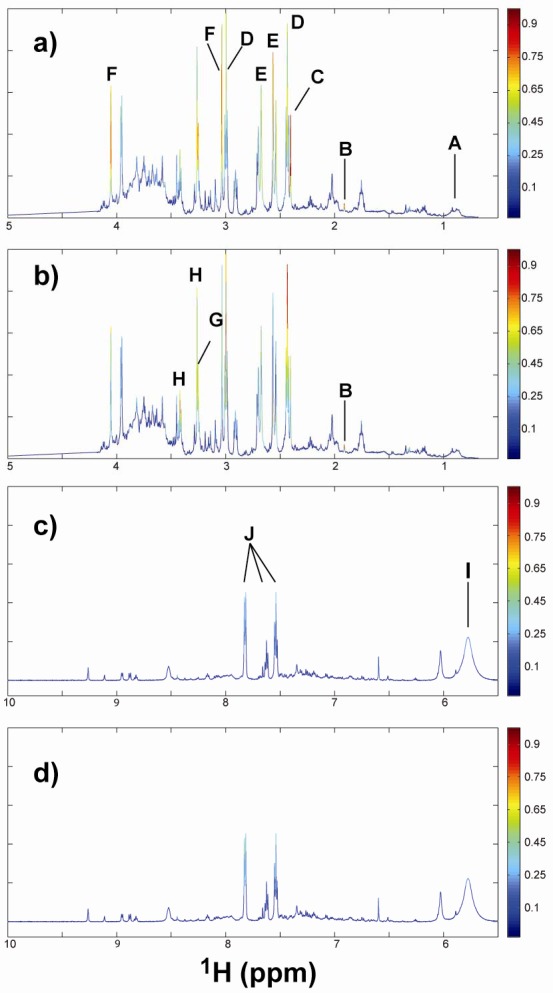
Urine ^1^H NMR spectra with color mapping showing the relative orthogonal signal correction-filtered partial least squares discriminate analysis (OSC-PLSDA) variable importance in projection (VIP) coefficients for sample classification. Representative spectrum is for a control animal in week 1. Expansion of different spectral regions with the VIP color coding with the TBP-treated (**a,c**) and the TPP-treated (**b,d**). Representative metabolites are labeled: A: Dibutyl phosphate, B: Acetate, C: Succinate, D: 2-Oxoglutarate, E: Citrate, F: Creatine, G: Betaine, H: Taurine, I: Urea, J: Benzoate.

The spectral regions responsible for the classification of TBP or TPP exposure were identified using the variable importance in projections (VIP) coefficients obtained during OSC-PLSDA (See experimental section for definition of VIP, Equation 1). Spectral regions with high VIP coefficients are more important in providing class separation during analysis, while those with very small VIP coefficients provide little contribution to classification. VIP coefficients were obtained for each exposure class; control, TBP- and TPP-exposed. Mapping of these VIP coefficients onto the ^1^H NMR spectra is shown in Figure 4 (only the TBP- and TPP-exposed classes are shown), with the colors representing the scaled VIP scores observed for each spectral region, allowing the identification of important metabolites. While there are similarities between the VIP coefficients in each class, inspection of Figure 4 shows that indeed different spectral regions are employed during the classification process. It is important to note that it is not the spectral intensity reflecting the VIP scores (like would be seen in a loadings plot), but the color coding associated with each frequency. For example, in Figure 4 the acetate resonance at δ = +1.91 ppm has a very large VIP score (red), even though the peak intensity for that particular spectrum is small.

The ^1^H NMR spectral regions with the highest ten VIP coefficients obtained from the chronic OSC-PLSDA are summarized in Table 2. The top VIP-identified spectral regions identified during the previous acute exposure studied are also provided in Table 2 for comparison [22]. Identification of the metabolites responsible for resonances in these spectral regions was accomplished using the CHENOMX NMR Suite metabolite spectral library. Some of the important metabolites identified are noted in Figure 4, and in Table 2. There are a few spectral regions with high VIP scores that were not assigned due to the inability to uniquely identify or resolve spectral features in highly overlapped regions. 2D NMR experiments such as COSY, TOCSY or HMQC could be pursued to help in identification of these regions, but were not obtained for the current samples. For classification of TPP exposed animals, the spectral regions with the highest two VIP scores were δ = +3.04 and +3.25 ppm, which have been assigned to succinate and betaine, respectively. The remaining top five spectral regions identified from these VIP scores (Table 2) are assigned to the endogenous metabolites acetate and creatine. For the TBP-exposed animals the top two spectral regions identified were δ = +3.0 ppm and +2.43 ppm, corresponding to 2-oxoglutarate. The other important metabolites also identified for TBP exposure were acetate, betaine and taurine (Table 2). There are also several other metabolites that have intermediate VIP scores (0.25 to 0.6) that are incorporated into the developed model: these include succinate, citrate and creatine.

The variation of the different VIP scores for the dominant metabolites as a function of exposure week is shown in Figure S1 (supplemental material). It should be emphasized that there is no single metabolite that provides complete classification for chronic TBP or TPP exposure over the entire exposure study. Instead it is a combination of variation in several metabolite profiles that give rise to the classification. While there are large variations in the relative importance of the different metabolites, a few trends should be noted. For the classification of TBP and TPP exposure the VIP scores for acetate and succinate begin relatively low. With increased exposure time the VIP scores for these metabolites increase, becoming >0.3 after 4 to 8 weeks of exposure, and a maximum near week 14. The VIP classification scores for 2-oxoglutarate shows very high values between week 2 and week 8, then decreases while the acetate and succinate VIP scores become more important. While these classification dynamics with exposure time are interesting, it is important to recall that the milestone of this paper was to identify metabolites that could be used for exposure classification. For this reason, the analysis of the entire time series simultaneously is the focus of our results shown in Table 2.

#### 2.3.1. Comparison of Metabolites for Acute and Chronic Exposure

Table 2 summarizes the important metabolites and corresponding VIP scores for both acute and chronic TBP or TPP exposure. The top three metabolites have been color coded for easy comparison between the different exposure classes. Many of the identified metabolites are similar for both acute and chronic classes, with differences in the relative ranking of importance. The chronic exposure does uniquely identify acetate as being important for both TBP and TPP classification, and was not previously observed during the acute exposure studies. Inspection of Figure 1 reveals this metabolite produces the sharp spectral signature (δ = +1.91 ppm) that appears in the later weeks of exposure, but then disappears again by week 15.

**Table 2:** Identification of important spectral regions and metabolites based on the VIP scores and OSC-PLSDA classification following both acute [22], and chronic organophosphate exposure. The top five VIP ranked metabolites are listed in parenthesis, with the top three for identification of each class color coded for quick comparison.

Resonance (ppm)	Metabolite	Scaled VIP Coefficients						
		Acute Exposure[22]				Chronic Exposure		
		Control	TBP	TPP		Control	TBP	TPP
0.91	Dibutyl Phosphate	<0.01	0.03	0.02		<0.01	<0.01	<0.01
1.31,1.32	Lactate	--	--	--		0.19	0.18	0.05
1.91	Acetate	--	--	--		1.00 (#1)	0.85 (#3)	0.65 (#5)
2.05	--	0.05	0.13	0.09		0.02	0.03	<0.01
2.40	Succinate	0.19	0.17	0.22		0.67	0.22	0.97 (#2)
2.41	Succinate	<0.10	0.18	0.25		0.33	0.05	0.95 (#4)
2.43	2-Oxoglutarate	0.37 (#5)	0.73 (#3)	0.52 (#4)		0.81 (#5)	0.94 (#2)	0.32
2.54, 2.56	Citrate	0.28	1.00 (#1)	0.45		0.33	0.24	0.46
2.68, 2.71	Citrate	0.23	0.80 (#2)	0.51 (#5)		0.17	0.20	0.20
3.00	2-Oxoglutarate	0.40 (#4)	0.63 (#4)	0.60 (#3)		0.83 (#4)	1.00 (#1)	0.25
3.04	Creatine	0.19	0.28 (#5)	0.25		0.93 (#2)	0.29	0.96 (#3)
3.25	Betaine	0.42 (#3)	0.17	0.50		0.89 (#3)	0.35 (#5)	1.00 (#1)
3.27	Taurine	0.82 (#2)	0.14	0.84 (#2)		0.30	0.27	0.31
3.41	--	0.33	<0.1	0.34		0.10	0.12	0.06
3.42	Taurine	1.00 (#1)	<0.1	1.00 (#1)		0.31	0.38 (#4)	0.15
3.43	--	0.25	<0.1	0.25		0.06	0.07	<0.01
3.67	--	0.13	0.16	0.17		<0.01	<0.01	<0.01
3.81	--	0.13	0.19	0.17		<0.01	<0.01	<0.01
4.06	Creatine	0.20	0.12	0.22		0.59	0.33	0.46
5.78	Urea	0.03	0.01	0.03		0.01	0.01	0.02
6.60	Fumarate	0.06	0.03	0.07		0.01	0.01	0.03
7.54	Benzoate	0.01	0.09	0.04		0.10	0.07	0.05
7.67	Benzoate	0.01	0.09	0.03		0.11	0.08	0.06
7.82, 7.83	Benzoate	0.01	0.10	0.04		0.11	0.07	0.06
8.44	Formate	--	--	--		0.10	0.05	0.13

### 2.4. Identification of Impacted Metabolite Pathways

**Figure 5:**
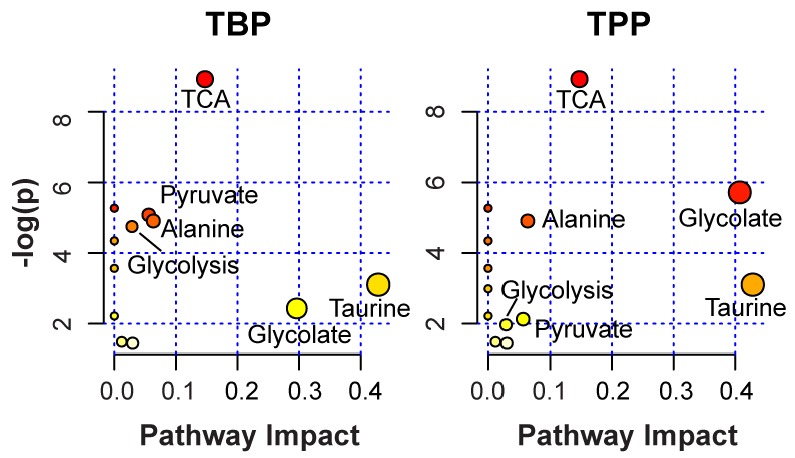
Metabolome view following metabolite pathway mapping of the impacted metabolites identified during chronic TBP or TPP exposure. The analysis was performed using the MetaboAnalyst software.

To determine what metabolic pathways were being impacted by chronic exposure to TBP and TPP, the metabolites with VIP scores > 0.1 (Table 2) were mapped using the MetaboAnalyst 2.0 software (www.metaboanalyst.ca/MetaboAnalysts). For analysis, the rat (*Rattus norvegicus*) pathway library and the hypergeometric test and the out-degree centrality algorithms were employed. The software provided a fit coefficient (p) from pathway enrichment analysis and an impact factor from pathway topology analysis for each analyzed pathway. The mapping of 14 different metabolic pathways is shown in Figure 5, with the top five pathways for TBP or TPP exposure being summarized in Table 3. While the metabolite list employed (Table 2) is rather limited and provides only two or three metabolite hits for each pathway, the mapping does allow a ranking of the relative importance and identification of different possibilities. The citrate cycle (TCA cycle) was identified as having the highest –log(p) value for both TBP and TPP chronic exposure. The TCA pathway involves changes in the cellular energy metabolism, and was previously identified as an impacted metabolic pathway for acute TBP and TPP exposure. Mapping to the glyoxylate and dicarboxylate pathway is also indicated as relatively important following chronic TPP exposure, and is related to the TCA cycle. The other identified pathways include the alanine, aspartate and glutamate metabolism, pyruvate, taurine and hypotaurine, and glycolysis metabolic pathways. This is consistent with the perturbation of the creatine production in the liver of roaches exposed to the OP pesticide fenitrothion, reported using non-targeted ^1^H NMR metabolomic studies [33]. Creatine synthesis is initiated in the kidney, and then completed in the liver. These same fenitrothion studies also revealed perturbations of the phenylalanine and tyrosine metabolite levels. While OP exposure is known to impact cellular metabolism in a variety of different tissues [34], the observed metabolite perturbations following chronic TBP and TPP exposure involved generic metabolic pathways, and does not provide information concerning the specific mechanisms of toxicity or targeted organs. Additional detailed organ or tissue specific studies would be required to address these chronic effects. 

**Table 3:** Metabolic pathway mapping of the important metabolites identified for chronic TBP and TPP exposure obtained using the MetaboAnalyst software.

		TBP			TPP		
Pathway Name	#	#			#		
	Metabolites	Hits	-log(p)	Impact	Hits	-log(p)	Impact
Citrate Cycle (TCA cycle)	20	3	8.92	0.15	3	8.92	0.15
Glyoxylate/Dicarboxylate	16	1	2.41	0.30	2	5.72	0.41
Taurine and Hypotaurine	8	1	3.10	0.43	1	3.10	0.43
Pyruvate	22	2	5.08	0.06	1	2.13	0.06
Alanine/Aspartate/Glutamate	24	2	4.91	0.06	2	4.91	0.06

## 3. Experimental Section

### 3.1. Animal Studies

The chronic exposure studies were performed on male Sprague-Dawley rats weighing 200-220 g (Harlan Sprague–Dawley Inc., Indianapolis, IN, USA) which were acclimatized for two weeks prior to the first dose in the animal care facility at the University of Texas Medical Branch (UTMB) Galveston. Seventeen rats were divided into three exposure classes: tributyl phosphate (TBP) exposure (5 rats), triphenyl phosphate (TPP) exposure (5 rats), and a control group (7 rats). Tributyl phosphate (TBP- 98.0% purity, Sigma Aldrich, USA) was dissolved in 1 ml corn oil and was administered by gavage to the rats using a daily 1.5 mg/kg body weight dose, while the TPP (98.0% purity, Sigma Aldrich, USA) was administrated daily at a 2.0 mg/kg body weight dose. The control rats received 1 mL of corn oil only. This dosing regime was repeated for a total of 15 weeks (Monday through Friday). On each Friday, the rats were transferred overnight to metabolic cages, with the urine collected Saturday afternoon (~20–22 hours after last weekly dose), and stored at -80 °C for further analysis. The rats were then returned to a normal cage for the following dose week. The 5 day dose, 2 days non-dose regime was used to reflect possible occupational exposure. The proton (^1^H) NMR data was only collected for urine samples from weeks 1, 2, 4, 8, 12, 14 and 15. The NMR spectra for one sample in week eight was corrupted during acquisition and was not included in the data set, with the total data set including 118 NMR spectra.

### 3.2. NMR Studies

The NMR analysis was performed on samples obtained from mixing 100 µl of urine with 650 µl of phosphate buffer giving a final concentration of 50 mM phosphate (pH = 6.0), 10% D_2_O, containing 500 µM DSS (2,2-dimethyl-2-silapentane-5-sulfonic acid) as a chemical shift indicator. The ^1^H NMR spectra were obtained using a Varian Unity Plus 600 with a three channel ^1^H-^13^C-^15^N (HCN) 5mm probe at 25 °C. A standard 1D NOESY pulse sequence, with a 1s recycle delay, a 1s water pre-saturation, 4 dummy scans, 256 scan averages, a 6 µs π/2 pulse width and a 100 ms mixing time (τ_m_) was employed. A spectral width of 20 ppm, with 28k complex data points, zero-filling to 64k points prior to Fourier transformation, and apodization using a 0.5 Hz exponential line broadening was used for all experiments. The NMR spectra were transformed, phased, chemical shift referenced (DSS δ = 0 ppm), and baseline corrected using CHENOMX NMR 7.0 Suite (Edmonton, Canada). The processed NMR data was binned to 0.001 ppm sections prior to analysis. This retains the spectral fine structure which is important during the variable importance in projection (VIP) score analysis described below. No peak alignment algorithms were employed on these datasets. The water region (4.2 to 5.5 ppm) was removed using the CHENOMX Suite prior to analysis. Due to the non-quantitative signal intensity resulting from the proton exchange between water and urea (which varies with the performance of water saturation), the urea spectral region (δ = 4.50 to 5.98 ppm) is commonly removed. In the present study, the urea spectral region was retained as it did not make a major impact on the PLSDA analysis. This impact is most easily seen in Figure 4, where the VIP score of urea remains relatively low (~0.25), is not a critical spectral region for classification. 

The processed NMR spectra were transferred at full resolution (no binning) for analysis in MATLAB2010b (The Mathworks) using PLS Toolbox 6.7 (Eigenvector Research, Inc.). The data sets were normalized using the Probabilistic Quotient Normalization (PQN) method [35] followed by mean centering. The PQN normalized data gave a small improvement in the observed cross-validation errors in comparison to integral normalization or constant sum normalization [36], and was used for all of the analysis presented here. 

### 3.3. Chemometric Analysis

This orthogonal signal correction (OSC) filtered partial least squares discriminate analysis (OSC-PLSDA) method has also been previously described [32,37], and was used to classify exposure in this NMR data set. The OSC-PLSDA method attempts to identify what spectral variations contribute to the identification of the designated classes. Orthogonal signal correction (OSC) was applied to remove non-correlating spectral variations (2 components) that were not contributing to classification. The OSC filtering can be integrated directly into the regular PLS-DA modeling, allowing the orthogonal variations to be analyzed separately [30,38,39]. This extended method is commonly referred to as O-PLSDA and should not be confused with the pre-filtering OSC-PLSDA method employed for the current analysis. Cross validation was performed using a Venetian Blind process, with the number of data splits equal to the nearest integer of the square root of the total number of samples in the data set. This number changed when a subset of weeks was analyzed. The VIP scores [40] were obtained from the OSC-PLSDA analysis and mapped onto the original NMR spectra. VIP coefficients reflect the importance of each spectral frequency to each variable in the PLS model. The VIP coefficient for the *k-*th parameter (frequency) is the sum over all PLS dimensions (*a*) of the contribution VIN (variable influence)


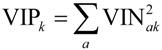
(1)

where 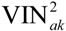 is equal to the squared PLS weight of that parameter multiplied by the percent explained sum of squares for that PLS dimension. 

## 4. Conclusions

These results demonstrate that ^1^H NMR metabolomics can be used to identify rats that have had long term chronic exposure to particular organophosphates; either tributyl phosphate or triphenyl phosphate. Using OSC-PLSDA chemometric modeling it was possible to classify and separate TBP-treated versus TPP-treated animals based on the NMR spectra of the urine. Unfortunately, the developed models were slightly disappointing requiring greater than twelve latent variables in order to keep the classification error below 10% under cross-validation analysis. A set of metabolites that were important for chronic TBP or TPP exposure classification were identified, and show some unique impacted metabolites in comparison to the set of metabolites obtained from the analysis of acute TBP or TPP exposure. These results demonstrate that metabolite response to environmental chemicals can provide a signature for identification of exposure.

## Supplementary Files

Supplementary File 1 PDF-Document (PDF, 114 KB)
