# Supplementary material for: 1H Nuclear Magnetic Resonance (NMR) Metabolomic Study of Chronic Organophosphate Exposure in Rats
Source: Metabolites. 2012 Jul 24;2(3):479–95. doi: 10.3390/metabo2030479 (PMC3901221; doi:10.3390/metabo2030479)

Supplemental Material

^{1}H NMR Metabolomic Study of Chronic Organophosphate Exposure in Rats

Todd M. Alam, Muniasamy Neerathilingam, M. Kathleen Alam, David E. Volk, G. A. Shakeel Ansari, Swapna Sarkar and Bruce A. Luxon

Figure S1: The evolution of the VIP scores for selected metabolites following OSC-PLSDA analysis on individual weeks. The VIP scores were determined for classification of control, TBP and TPP chronic exposure.

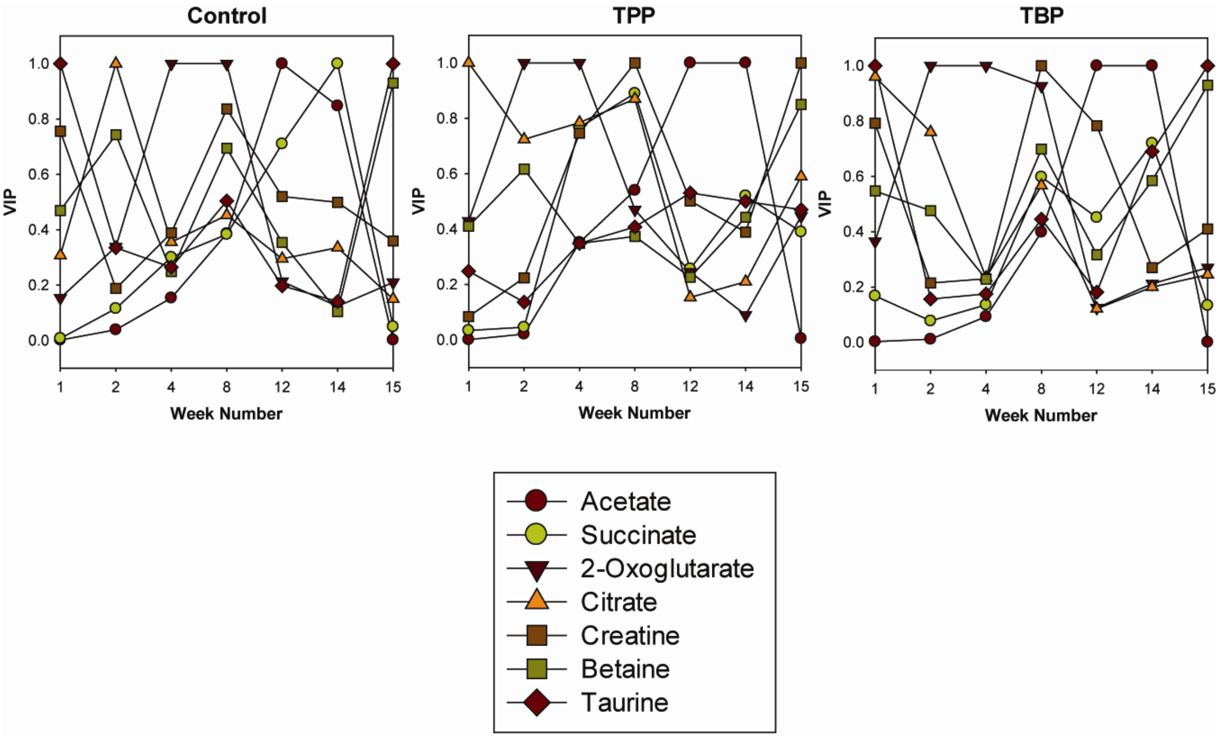

Supplement: Supplementary File 1 — PDF-Document (PDF, 114 KB) [file metabolites-02-00479-s001.pdf]
